# Synthesis and Characterization of Lithium Pyrocarbonate (Li_2_[C_2_O_5_]) and Lithium Hydrogen Pyrocarbonate (Li[HC_2_O_5_])

**DOI:** 10.1002/anie.202409822

**Published:** 2024-11-06

**Authors:** Dominik Spahr, Lkhamsuren Bayarjargal, Maxim Bykov, Lukas Brüning, Pascal L. Jurzick, Victor Milman, Nico Giordano, Mohamed Mezouar, Björn Winkler

**Affiliations:** ^1^ Goethe University Frankfurt Institute of Geosciences Altenhöferallee 1 60438 Frankfurt Germany; ^2^ Goethe University Frankfurt Institute of Inorganic and Analytical Chemistry Max-von-Laue-Straße 7 60438 Frankfurt Germany; ^3^ University of Cologne Institute of Inorganic Chemistry Greinstraße 6 50939 Cologne Germany; ^4^ Dassault Systèmes BIOVIA 22 Cambridge Science Park Cambridge CB4 0FJ United Kingdom; ^5^ Deutsches Elektronen-Synchrotron DESY Notkestrasse 85 22607 Hamburg Germany; ^6^ European Synchrotron Radiation Facility ESRF 71 avenue des Martyrs CS40220, 38043 Grenoble Cedex 9 France

**Keywords:** symmetric hydrogen bond, pyrocarbonate, hydrogen pyrocarbonate, single crystal diffractio, high-pressure synthesis

## Abstract

The anhydrous pyrocarbonate and the first hydrogen pyrocarbonate Li[HC_2_O_5_] have been synthesized in a laser‐heated diamond anvil cell at moderate pressures (≈25 GPa). The structures of the two compounds have been obtained from single crystal X‐ray diffraction data. Raman spectroscopy and DFT calculations have been employed to further characterize their structure‐property relations. The present results significantly enlarge the group of inorganic pyrocarbonates by the discovery of the hydrogenated pyrocarbonate anion Li[HC_2_O_5_]^−^. In the structure of Li[HC_2_O_5_] there is a symmetric O−H−O arrangement at high pressures, which converts to a conventional O−H⋯ O hydrogen bond upon pressure release.

## Graphical abstract

Li[HC_2_O_5_] is the first hydrogen‐containing pyrocarbonate. It was synthesized by a reaction of CO_2_ and H_2_O with Li_2_[CO_3_] at moderate pressures and temperatures together with an anhydrous lithium pyrocarbonate (Li_2_[C_2_O_5_]) in a DAC. The crystal structure was determined by single crystal X‐ray diffraction and confirmed by DFT‐based calculations. The structure refinement and DFT calculations show that in Li[HC_2_O_5_] a symmetric hydrogen bond is present in the hydrogenated pyrocarbonate anion [HC_2_O_5_]^−^.

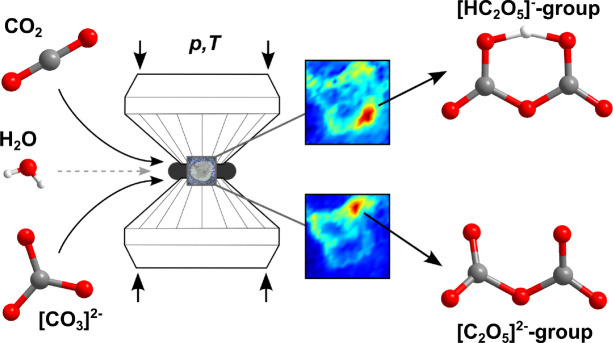

Carbonates are studied to address numerous questions in science and technology, as they are the major reservoir of carbon in the biosphere, hydrosphere, in soils and in the Earth's crust.[^1^, ^2^] They are important constituents in a large variety of consumer products, and play a fundamental role in technological processes.[^3^, ^4^] For example, the mineral zabuyelite (Li_2_[CO_3_]) is an important intermediate product in the lithium extraction process from dried salt lakes.[^5^, ^6^]

In order to be able to establish comprehensive and predictive models for structure‐property relations of carbonates, it is necessary to understand their structures and stability fields as a function of pressure, temperature and composition by carrying out synthesis experiments. Conversely, predicted structural models can be benchmarked by such synthesis studies.

“Conventional” carbonates, such as Li_2_[CO_3_] or the geologically much more relevant Ca[CO_3_] are characterized by the presence of nearly planar trigonal carbonate anions (${\left[{\mathrm{CO}}_{3}\right]}^{2-}$ ), where the interatomic bonding involves C‐*sp* ^2^ hybrid orbitals between the central carbon atom and the three surrounding oxygen atoms.[^7^, ^8^, ^9^] These carbonates are the anhydrous salts of the carbonic acid (H_2_[CO_3_]), and the isolated ${\left[{\mathrm{CO}}_{3}\right]}^{2-}$ ‐groups are not connected to each other.

Recently, the synthesis of Sr[C_2_O_5_] and isostructural Pb[C_2_O_5_] established inorganic anhydrous pyrocarbonate salts as a new family of carbonates.[^10^, ^11^, ^12^] Inorganic anhydrous pyrocarbonates can be considered to be the salts of the hypothetical pyrocarbonic acid (H_2_[C_2_O_5_]). In these pyrocarbonates two ${\left[{\mathrm{CO}}_{3}\right]}^{2-}$ ‐groups are connected by sharing one oxygen atom, resulting in the formation of a ${\left[C_{2}O_{5}\right]}^{2-}$ ‐anion. In the last two years, several inorganic anhydrous pyrocarbonates have been synthesized at moderately high pressures (≈20 −40 GPa), and pyrocarbonates with mono‐, di‐, and trivalent metal cations have been obtained.[^10^, ^11^, ^13^, ^14^, ^15^] The ${\left[C_{2}O_{5}\right]}^{2-}$ ‐anion is rather flexible due to the rotational degree of freedom around the bridging oxygen atom and hence can adapt itself to a broad range of structural environments. It therefore now seems plausible that inorganic pyrocarbonates may be obtained for all established “conventional” carbonates, and in fact may be the predominant carbonate phases at moderate pressures and high CO_2_‐fugacities.

Partial deprotonation of H_2_[CO_3_] leads to an intermediate hydrogenated anion containing one hydrogen atom. The corresponding salts are called hydrogencarbonates (colloquially known as bicarbonates). In these carbonates one of the oxygen atoms of the ${\left[{\mathrm{CO}}_{3}\right]}^{2-}$ ‐group is connected to a hydrogen atom forming a [HCO_3_]^−^‐group. Crystal structures of hydrogencarbonates such as Na[HCO_3_], K[HCO_3_] or Cs[HCO_3_] are well established,[^16^, ^17^, ^18^] but Li[HCO_3_] has not yet been demonstrated to exist in solid form,^[19]^ i.e. there is no report of a single crystal diffraction study of Li[HCO_3_]. There is no obvious reason why hydrogenated pyrocarbonate anions ([HC_2_O_5_]^−^) should not exist in analogy to conventional hydrogencarbonates, but they have neither been predicted nor synthesized up to now.

In the present study we investigated the reaction of Li_2_[CO_3_] with CO_2_ between 10 GPa and 25 GPa and at elevated temperatures in order to obtain an inorganic lithium pyrocarbonate salt. The high‐pressure experiments were carried out in laser‐heated diamond anvil cells (LH‐DACs). Li_2_[CO_3_] powder was compacted between a diamond and a glass plate. In a second step, the powder compact was placed on the culet of the lower diamond of the DAC and a ruby chip for pressure determination was added. Afterward, the DAC was cooled down to ≈100 K for the cryogenic loading. CO_2_‐I (dry ice) was directly condensed into the gasket hole from a CO_2_ gas jet until the gasket hole and the powder compact were completely covered. In the last step, the DAC was tightly closed and compressed to the target pressure without intermediate heating. While we use argon as a purge gas, sometimes the co‐condensation of H_2_O‐ice cannot be completely prevented (see SI).

During cold compression CO_2_‐I ($Pa\bar{3}$ ) undergoes a pressure‐induced phase transition to CO_2_‐III (*Cmca*) in a broad (≈5 GPa) pressure range around ≈12 GPa.[^20^, ^21^] The experimental Raman spectrum of CO_2_‐III at 25(2) GPa is accurately reproduced by the Raman spectrum obtained from the DFT‐based calculations (Figure 1 a). Heating of CO_2_ at relatively low pressures causes the appearance of high‐temperature polymorphs such as phase II or IV (see summary in Ref. [22]).[^23^, ^24^] At ambient conditions Li_2_[CO_3_] crystallizes in space group C2/c .^[25]^ Upon compression a phase transition from *γ*‐Li_2_[CO_3_] to a Li_2_[CO_3_]‐*P*6_3_ */mcm* phase was found experimentally at ≈10 GPa and the hexagonal phase was predicted to be stable at >8 GPa by DFT‐based calculations.[^26^, ^27^] The experimentally obtained Raman spectrum of Li_2_[CO_3_] at 25 GPa prior to the laser heating is in agreement with the Raman spectrum from our DFT‐based calculations in the high‐pressure space group *P*6_3_ */mcm* (Figure 1 b). In summary, the contributions from all phases in the DAC to the experimental Raman spectra before the laser‐heating are well understood.

**Figure 1:**
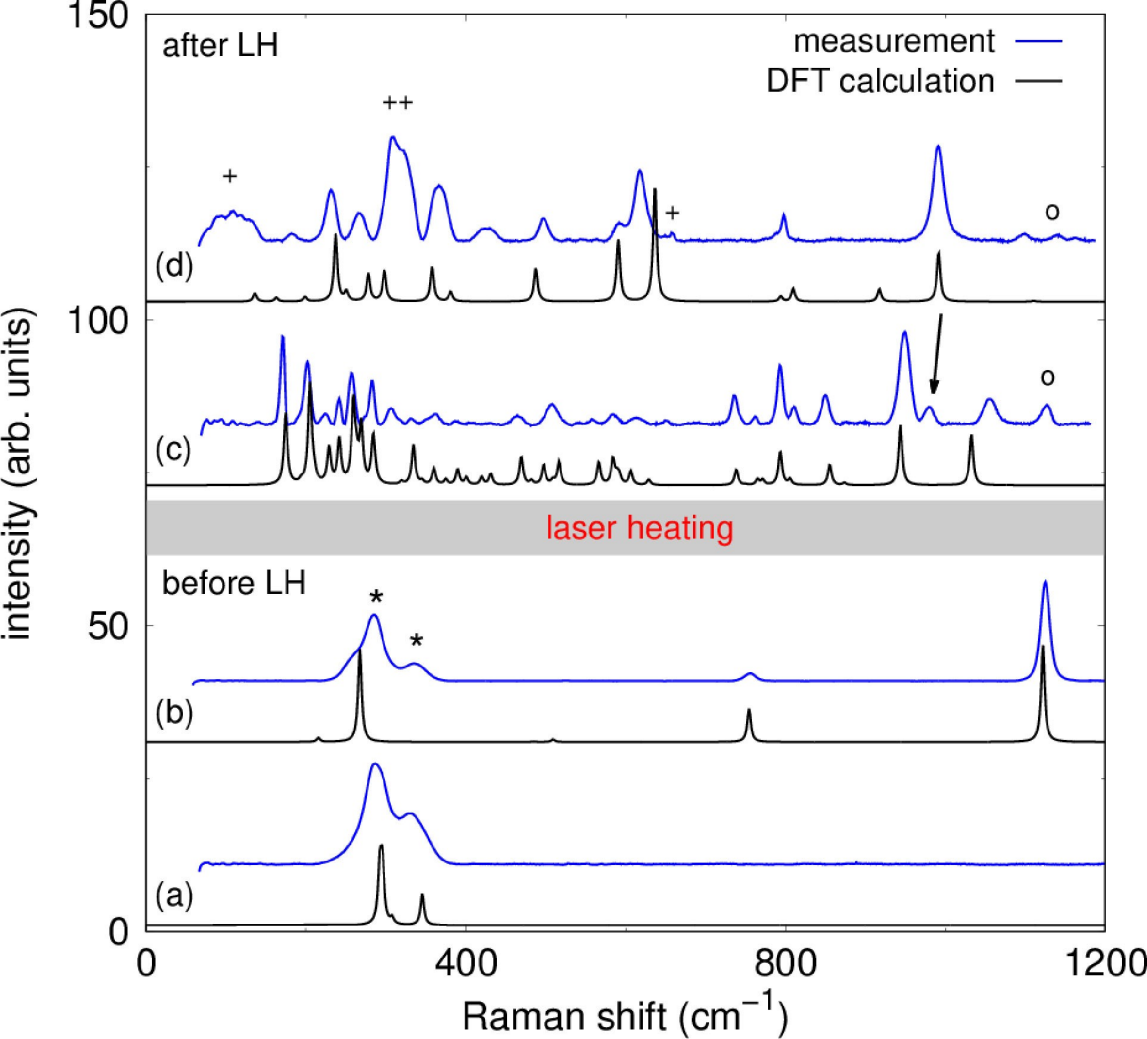
(a) Raman spectra for CO_2_‐III at 25(2) GPa. (b) Raman spectra for the high‐pressure phase Li_2_[CO_3_]‐*P*6_3_ */mcm* at 25(2) GPa. (c) Raman spectra of Li_2_[C_2_O_5_] after the synthesis at elevated pressures and temperatures. (d) Raman spectra of Li[HC_2_O_5_] after the synthesis. Experimental Raman spectra are shown in blue and DFT‐based calculations (rescaled by 2 %) are shown in black. Peaks of CO_2_‐III are marked by an asterisk (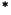 ), of Li_2_[CO_3_] by a circle (∘ ) and of CO_2_‐IV by a cross (+). The arrow indicates the position of the strongest Raman mode of Li[HC_2_O_5_].

The Li_2_[CO_3_]+CO_2_ mixture was laser‐heated from both sides at pressures between 10 GPa and 25 GPa in several experiments. In this pressure range the direct and indirect heating of CO_2_‐III results in a phase transformation into CO_2_‐IV, causing the appearance of strong new Raman modes at low wavenumbers (<400 cm^−1^).^[24]^ We found that heating Li_2_[CO_3_] in the CO_2_ atmosphere at pressures ≥20 GPa causes the appearance of new Raman modes in the region between 700 cm^−1^ and 1100 cm^−1^ at ambient temperatures, which are characteristic for vibrations of ${\left[C_{2}O_{5}\right]}^{2-}$ ‐groups.[^10^, ^11^, ^13^, ^14^, ^15^]

Heating the sample for 30 minutes to a maximum temperature of $\approx 1500\left(200\right)$ K at 25(2) GPa (Figure 2 a) resulted in an ambient temperature Raman spectrum of the unknown phase with very little contamination by other phases (Figure 1 c). When mapping the intensities of the Raman modes of the unknown phase across the gasket hole we found that a second new and unknown phase is present (Figure 2 b–d). The second phase also shows characteristic vibrations for ${\left[C_{2}O_{5}\right]}^{2-}$ ‐groups (Figure 1 d). In addition CO_2_‐IV is present in the heated areas (Figure 2e), while CO_2_‐III (Figure 2 f) only occurs at the borders of the gasket hole. The Raman maps allowed us to determine those locations in the gasket hole with the highest concentrations of the two new phases. These regions (Figure 2 b‐d) were then chosen for subsequent single crystal X‐ray diffraction experiments using a μm‐sized X‐ray beam (see SI).

**Figure 2:**
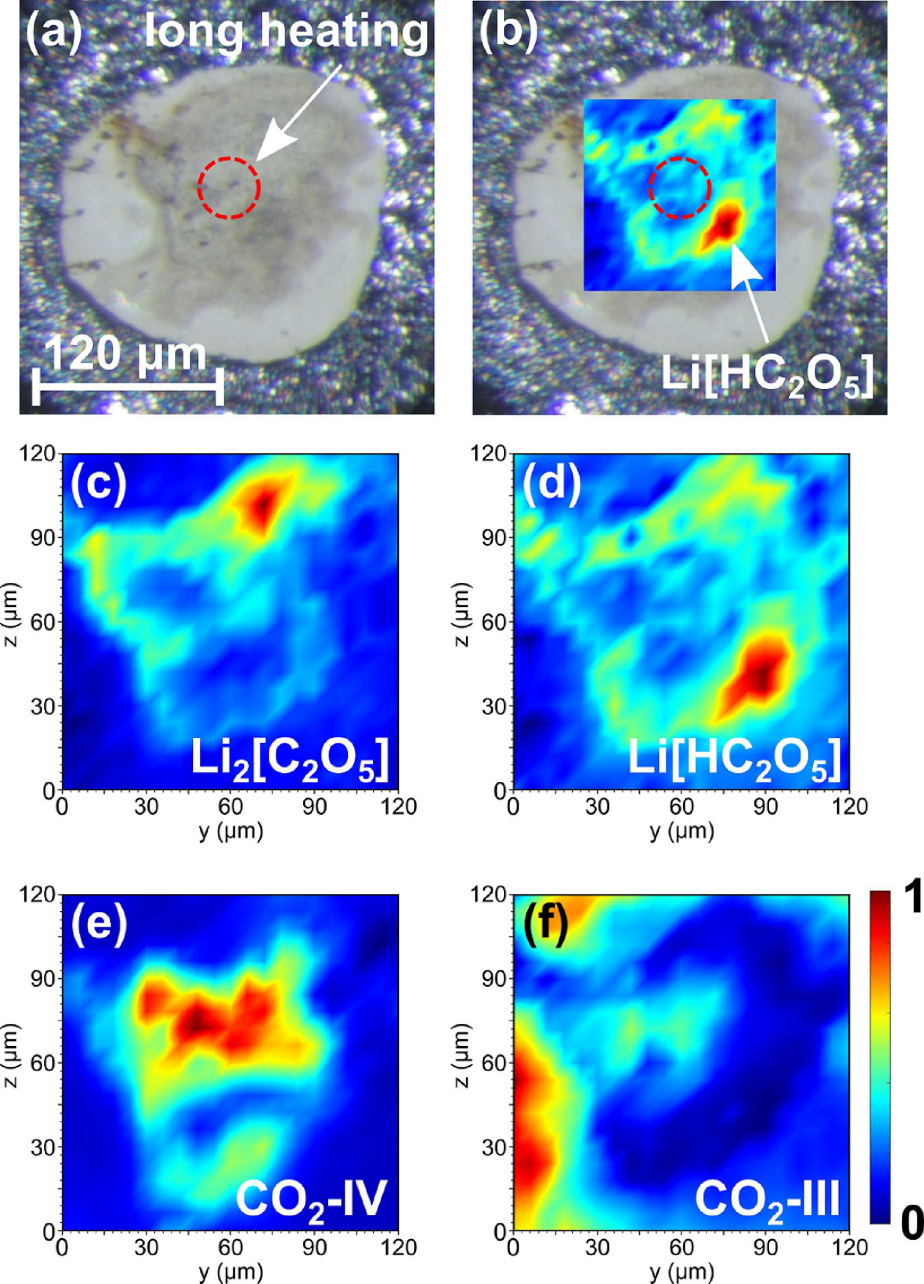
(a) The Li_2_[CO_3_]+CO_2_ mixture after laser heating at 25(2) GPa up to temperatures of $\approx 1500\left(200\right)$ K. (b) Raman map for Li[HC_2_O_5_] as an overlay over the photograph of the gasket hole. Raman maps of: (c) Li_2_[C_2_O_5_], (d) Li[HC_2_O_5_], (e) CO_2_‐IV and (f) residual CO_2_‐III.

We determined the crystal structure of the first unknown phase and found that it is the inorganic pyrocarbonate salt Li_2_[C_2_O_5_] (Figure 3 a). Li_2_[C_2_O_5_] crystallizes at 25(2) GPa in the monoclinic space group *P*2_1_ */c* with Z=4 and $a=6.085\left(1\right)$ Å, $b=5.313\left(3\right)$ Å, $c=7.996\left(3\right)$ Å and $\beta =100.85{\left(3\right)}^{\circ }$ ($V=253.9\left(2\right)$ Å^3^). The crystal structure is characterized by the presence of isolated ${\left[C_{2}O_{5}\right]}^{2-}$ ‐groups without any residues attached to the oxygen atoms. The agreement between the experimental structure refinement and the results from our DFT‐based full geometry optimizations is very good (see Table S1). In addition, the DFT‐calculated Raman spectrum nicely reproduces the experimental data (Figure 1 c). Hence, the structure of Li_2_[C_2_O_5_] at 25(2) GPa is now unambiguously established, showing that for yet another “conventional” carbonate a corresponding pyrocarbonate can be obtained. During the review of the present manuscript, Sagatova *et al*.^[28]^ published a crystal structure prediction of Li_2_[C_2_O_5_]. The predicted structure does not agree with the structre found experimentally by us, as it has a different space group ($P\bar{1}$ ) instead of *P*2_1_ */c*, and the topology is different.

**Figure 3:**
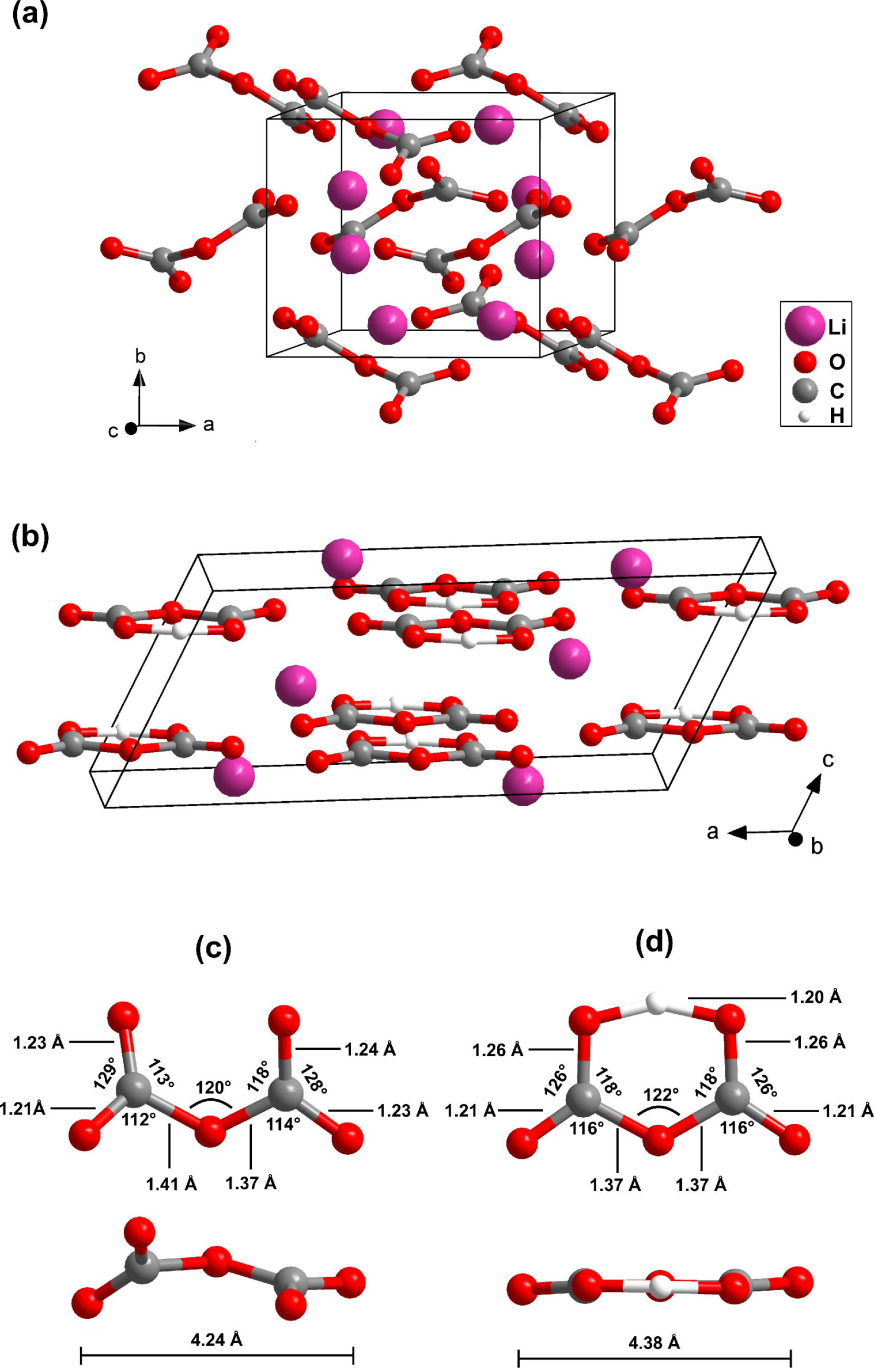
(a) Monoclinic structure (*P*2_1_ */c*, Z=4 ) of lithium pyrocarbonate (Li_2_[C_2_O_5_]). (b) Monoclinic structure (C2/c , Z=4 ) of lithium hydrogen pyrocarbonate (Li[HC_2_O_5_]). (c) Geometry of the ${\left[C_{2}O_{5}\right]}^{2-}$ ‐groups in Li_2_[C_2_O_5_]. (d) Geometry of the [HC_2_O_5_]^−^‐groups in Li[HC_2_O_5_]. Structural models were obtained by single crystal diffraction at 25(2) GPa. The experimental uncertainties are: ≈0.003 Å (O−C bonds), ≈0.01 Å (O−H bond) and $\approx 0.3^{\circ }$ (angles).

The crystal structure of the second unknown phase was also solved and refined from single crystal X‐ray diffraction data. We found that this phase is a hydrogen pyrocarbonate, Li[HC_2_O_5_] (Figure 3 b). Li[HC_2_O_5_] crystallizes at 25(2) GPa in the monoclinic space group C2/c with Z=4 and $a=12.085\left(9\right)$ Å, $b=4.373\left(1\right)$ Å, $c=5.231\left(7\right)$ Å and $\beta =117.5{\left(1\right)}^{\circ }$ ($V=245.3\left(4\right)$ Å^3^). Due to the presence of only light atoms the hydrogen atom could easily be recognized in the difference Fourier map. A refinement without a hydrogen atom results in a strong residual electron density between the two oxygen atoms (Figure 4 a). After introducing the hydrogen atom of the [HC_2_O_5_]^−^‐group, the residual electron density between the two oxygen atoms vanishes (Figure 4 b) and the *R*‐value decreases by ≈0.5 %. The experimental error in the hydrogen position is larger than for the other atoms. However, it is generally accepted that DFT model calculations can reliably predict hydrogen positions.^[29]^ The experimental structural model for Li[HC_2_O_5_] is fully supported by the DFT‐based calculations, and so it is now well established that in this hydrogen pyrocarbonate a symmetric hydrogen bonding occurs within the [HC_2_O_5_]^−^‐group at 25(2) GPa (see Table S2). The planar [HC_2_O_5_]^−^‐group exhibits *C*_2_ point symmetry because it lies on a special position.

**Figure 4:**
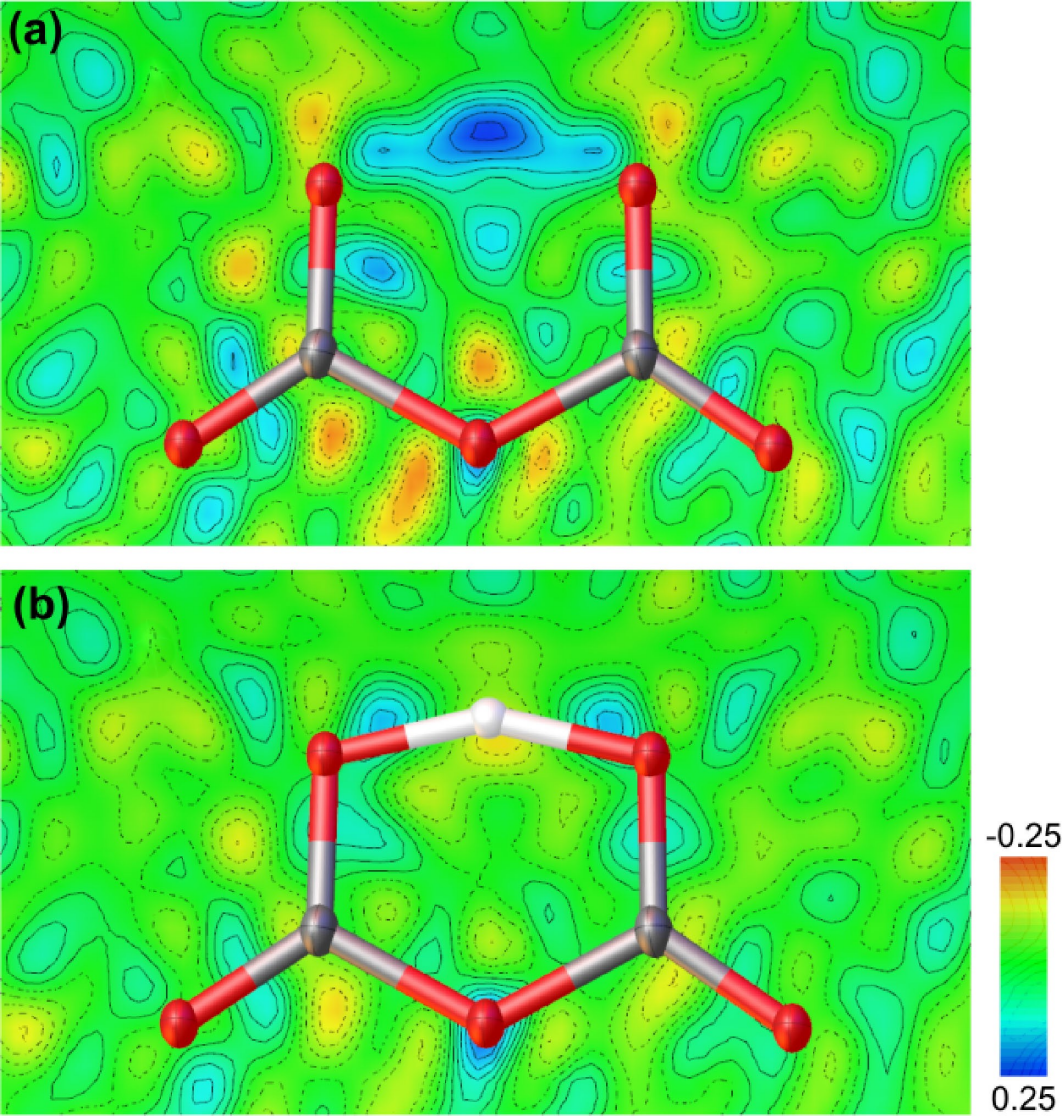
Difference Fourier map arround the [HC_2_O_5_]^−^‐group in Li[HC_2_O_5_] at 25(2) GPa: (a) refinement without a hydrogen and (b) with a hydrogen atom placed between the oxygen atoms.

Figures 3 (c) & (d) show the geometry of the ${\left[C_{2}O_{5}\right]}^{2-}$ ‐groups in Li_2_[C_2_O_5_] in comparison to the geometry of the [HC_2_O_5_]^−^‐groups in Li[HC_2_O_5_]. The bond lengths and bond angles, other than the torsion between the two [CO_3_]^2−^‐groups in the [C_2_O_5_]^2−^‐groups, are very similar between the pyro‐groups with and without the hydrogen. Whether or not the hydrogen bond causes the [HC_2_O_5_]^−^‐group to be planar is currently unknown, as planar [C_2_O_5_]^2−^‐groups also occur in anhydrous pyrocarbonates.^[14]^ The geometries of the pyrocarbonate groups in Li_2_[C_2_O_5_] and Li[HC_2_O_5_] are also similar to those of the ${\left[C_{2}O_{5}\right]}^{2-}$ ‐groups in other pyrocarbonates.[^10^, ^11^, ^13^, ^14^, ^15^]

Experimentally it is found that at 25(2) GPa a symmetric hydrogen bond is present in the [HC_2_O_5_]^−^‐group within the experimental uncertainty (Figure 3 d). This is supported by our DFT‐based calculations, which show that at pressures ≥10 GPa the hydrogen bond is symmetric (Figure 5 a). The O−H−O distances in the [HC_2_O_5_]^−^‐group are similar to those in the symmetrized hydrogen bond in *δ*‐AlOOH (≈1.2 Å) at 18 GPa.^[30]^ Symmetric hydrogen bonds have also been observed in the high‐pressure phase of H_2_O ice‐X at pressures ≥60 GPa.^[31]^

**Figure 5:**
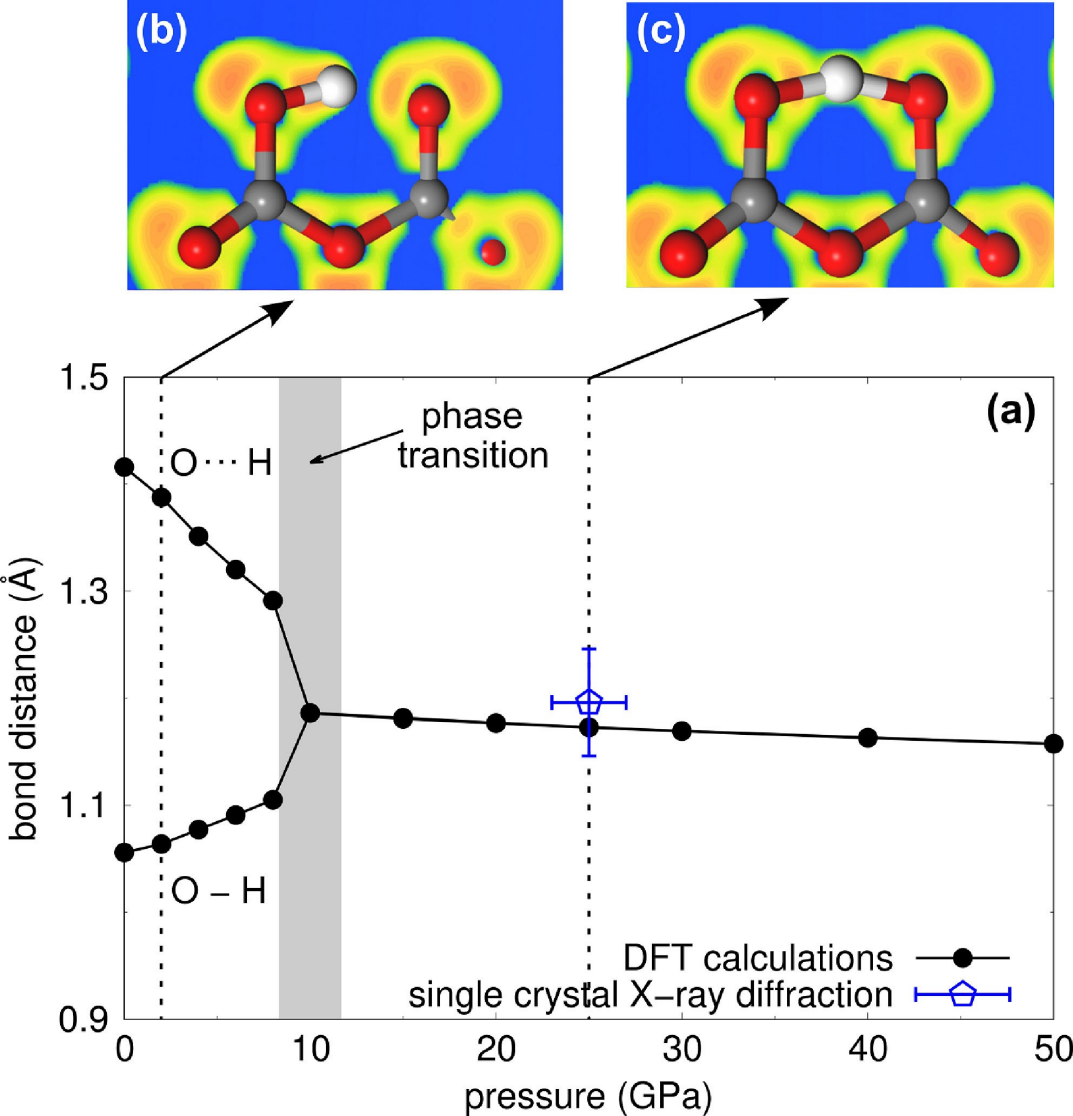
(a) Pressure dependent O−H and O⋯H bond lengths in the [HC_2_O_5_]^−^‐group of Li[HC_2_O_5_]. Electron localization function from DFT calculations of the [HC_2_O_5_]^−^‐group at (b) 2 GPa and (c) 25 GPa, showing the transition from a asymmetric double‐well hydrogen bond to a single‐well symmetric hydrogen bond. The black line connecting the data points is a guide to the eye.

The DFT calculations for Li[HC_2_O_5_] were carried out in space group *Cc*. In this setting, there are no symmetry constraints on the hydrogen position. This allows us to investigate the behavior during pressure release. We found that at pressures <10 GPa the O−H−O system becomes a double‐well hydrogen bond and a clear distinction between the acceptor and the donor oxygen atom (O⋅⋅⋅H−O) can be made (Figure 5). The barrier between the double‐wells is very small (0.01 eV per unit cell at 8 GPa). Figures 5 (b) & (c) show the electron localization function^[32]^ from DFT calculations of the [HC_2_O_5_]^−^‐group at 2 GPa and at 25 GPa, demonstrating the symmetric O−H−O bonding at elevated pressures. At 2 GPa the covalent O−H bond has a Mulliken bond population of 0.49 e^−^/Å^3^, while the O⋅⋅⋅H bond has a population of 0.24 e^−^/Å^3^. In contrast the symmetric O−H−O bonds at 25 GPa both have Mulliken bond populations of 0.41 e^−^/Å^3^.

We used the unit cell volumes obtained by the DFT calculations to determine the compression mechanism and the bulk moduli of the hydrous lithium pyrocarbonate and anhydrous lithium pyrocarbonate phases. For Li[HC_2_O_5_] there is no noticeable dependence of the volume on the detailed configuration of the O−H−O group, i.e. the pressure‐induced symmetrization of the hydrogen bond does not change the compressibility (Figure S3). For the hydrogen pyrocarbonate, we obtain a bulk modulus of $K_{0}=25.7\left(4\right)$ GPa with *K*_p_=5.9(1). The calculations show that for this phase, a van der Waals correction to the standard DFT‐GGA‐PBE approach is required, as otherwise the pressure dependence of the unit cell volumes at low pressures cannot be described with a reasonable equation of state (EoS) (Figure S3). When a vdW‐correction is employed, the whole data set can be well represented by a single EoS‐fit and a fit between 0–50 GPa or 10–50 GPa will result in the same values for *K*_0_ and *K*_p_ (Table S3). For anhydrous Li_2_[C_2_O_5_], calculations at lower pressures imply that it would undergo a spontaneous deformation and that the high‐pressure phase cannot be recovered (Figure S4–S6). Hence, we used the *p,V* data ≥10 GPa for the determination of the bulk modulus, which can be well represented by a single EoS‐fit. We obtained a bulk modulus of $K_{0}=41\left(2\right)$ GPa with $K_{p}=5.9\left(1\right)$ for Li_2_[C_2_O_5_] from the EoS between 10–50 GPa, which is significantly larger than for Li[HC_2_O_5_].

In conclusion, we enlarged the family of carbonates by the synthesis of the first hydrogen pyrocarbonate Li[HC_2_O_5_] and anhydrous Li_2_[C_2_O_5_]. The present study therefore has not only strengthened the hypothesis, that pyrocarbonate‐analogs of all “conventional” carbonates can be obtained, but also demonstrated that hydrogen pyrocarbonates, such as Li[HC_2_O_5_] can be obtained, even if no “conventional” hydrogencarbonate analog, such as Li[HCO_3_], has been found yet. The first synthesis of a hydrogen pyrocarbonate yielded a structure with a symmetric O−H−O arrangement within the pyrocarbonate group. It is now of interest to understand, if this is typical or if hydrogen bonds between pyrocarbonate groups can be formed.

## Supporting Information

The supplementary material contains the experimental and computational details of the experiments. Furthermore, additional information to the results of the single crystal structure solution and DFT‐based calculations are shown. Experimental and DFT‐calculated structural data has been deposited at the Cambridge Crystallographic Data Centre (CCDC).^[33]^

## Conflict of Interests

There are no conflicts to declare.

## Supporting information

As a service to our authors and readers, this journal provides supporting information supplied by the authors. Such materials are peer reviewed and may be re‐organized for online delivery, but are not copy‐edited or typeset. Technical support issues arising from supporting information (other than missing files) should be addressed to the authors.

Supporting Information

## Data Availability

The supplementary material contains the experimental and computational details together with the crystallographic data associated with this article. Raw experimental data are available on request from the authors.
