# Supplementary material for: Synthesis and Characterization of Lithium Pyrocarbonate (Li2[C2O5]) and Lithium Hydrogen Pyrocarbonate (Li[HC2O5])
Source: Angew Chem Int Ed Engl. 2024 Nov 6;63(49):e202409822. doi: 10.1002/anie.202409822 (PMC11586701; doi:10.1002/anie.202409822)
Supplement: Supplementary file 1 — Supporting Information [file ANIE-63-e202409822-s001.pdf]

Angewandte Chemie
Eine Zeitschrift der Gesellschaft Deutscher Chemiker

Supporting Information

Synthesis and Characterization of Lithium Pyrocarbonate ($Li_2[C_2O_5]$) and Lithium Hydrogen Pyrocarbonate ($Li[HC_2O_5]$)

D. Spahr, L. Bayarjargal, M. Bykov, L. Brüning, P. L. Jurzick, V. Milman, N. Giordano, M. Mezouar, B. Winkler*

Supplementary material: Synthesis and Characterization of Lithium Pyrocarbonate ($Li_2[C_2O_5]$) and Lithium Hydrogen Pyrocarbonate ($Li[HC_2O_5]$)

Dominik Spahr[*,a], Lkhamsuren Bayarjargal[a], Maxim Bykov[b], Lukas Brüning[b], Pascal L. Jurzick[c], Victor Milman[d], Nico Giordano[e], Mohamed Mezouar[f], Björn Winkler[a]

[a]*Goethe University Frankfurt, Institute of Geosciences, Altenhöferallee 1, 60438 Frankfurt, Germany*
[b]*Goethe University Frankfurt, Institute of Inorganic and Analytical Chemistry, Max-von-Laue-Straße 7, 60438 Frankfurt, Germany*
[c]*University of Cologne, Institute of Inorganic Chemistry, Greinstraße 6, 50939 Cologne, Germany*
[d]*Dassault Systèmes BIOVIA, 22 Cambridge Science Park, Cambridge CB4 0FJ, United Kingdom*
[e]*Deutsches Elektronen-Synchrotron DESY, Notkestrasse 85, 22607 Hamburg, Germany*
[f]*European Synchrotron Radiation Facility ESRF, 71 avenue des Martyrs, CS40220, 38043 Grenoble Cedex 9, France*

1. Methods

1.1. *Sample material*

Commercial $Li_2[CO_3]$ powder (99.999% purity, Thermo Fisher Scientific, Waltham, USA) was used for the high-pressure experiments without further purification. Before the diamond anvil cell (DAC) loading the $Li_2[CO_3]$ powder was dried in an oven at 573(1) K for 12 h. Afterwards, the powder was compacted between a diamond and a glass plate to obtain a 10–20 μm thin powder compact. We used the CO_2 gas for the gas-jet (Nippon gases, purity $\geq$ 99.995%) and the argon purge gas (Nippon gases, purity $\geq$ 99.999%) as purchased.

1.2. *High-pressure experiments*

The high-pressure experiments were carried out using Boehler-Almax type DACs with 350 μm culet size.[1] Diamonds with 70° opening angle on the top and with 85° opening angle on the bottom were used for the experiments. Re-gaskets were pre-indented to a thickness of $\approx$ 45 μm and placed between the top and bottom diamonds. Gasket-holes with 140 μm diameter were drilled by a custom-built laser setup. In a first step the powder compact with dimensions of $\approx 80 \times 80$ μm^2 and a thickness of 10–20 μm was placed on the culet of the bottom diamond. Afterwards, we added a ruby chip for pressure determination in the gasket hole on the bottom diamond. The pressure was determined by measuring the shift of the ruby fluorescence and we assume an error of 6% due to non-hydrostatic conditions.[2] We assume that the pressure conditions in the DAC before laser-heating are very likely non-hydrostatic as CO_2-III may sustain pressure gradients up to 0.2 GPa μm^{-1} at high pressures without heating.[3]

The CO_2 (dry-ice) was directly condensed into the gasket hole using a custom-built cryogenic loading system (see Spahr *et al.*[4]) derived from an earlier concept.[5] The DAC was slightly opened and placed on a liquid nitrogen cooled Cu-holder and it was cooled down to $\approx$ 100 K. We used a small nozzle to align the CO_2 gas jet with 5 l min^{-1} directly on the gap between upper diamond and the gasket. Ar (10 l min^{-1}) was used as a purge-gas to avoid the precipitation of H_2O ice, but we assume that small amounts of H_2O were inadvertently co-condensed from the residual moisture in the CO_2- or Ar-gas. In the current study, the co-condensation of H_2O into the DAC occurred unintentionally. An improvement of the experimental set-up is planned, but it will remain practically impossible to co-condense specific quantities of different gases. The precipitation of the CO_2 in the gasket hole was monitored by an optical microscope and a camera. After a sufficient amount of CO_2 was gathered in the gasket hole, the DAC was tightly closed and compressed to the target pressure without intermediate heating.

1.3. *Raman spectroscopy and laser heating*

High-pressure Raman spectroscopy and the double-sided laser-heating in DACs were performed using a custom-built set-up.[6] Raman spectroscopy was performed with an Oxxius LCX-532S Nd:YAG laser (λ = 532.14 nm) in combination with a Princeton Instruments ACTON SpectraPro (SP-2356) spectrograph equipped with a Pixis 256E CCD camera. Applying a laser power of 250 mW on the sample, the spot size of the Raman laser was $\approx$ 6 μm. Raman maps were measured on a grid with a step-size of 6 μm in x- and y-direction. Afterwards, the background was corrected using the software package Fityk.[7]

Double-sided laser-heating was performed using a Coherent Diamond K-250 pulsed CO_2 laser (λ = 10600 nm). The laser power was adjusted to achieve a coupling of the laser to the sample using a laser power of $1-3$ W. Focusing on the sample results in a heating area of $\approx 40 \times 40$ μm^2 and the highest temperature achieved during the laser heating was $T_{max} \approx 1500(200)$ K. The temperatures were determined by the two-color pyrometer method, employing Planck and Wien fits.[8] The heating time was $\approx$ 30 min. It is well established that laser-heating in DACs always suffers from large temperature gradients and the actual temperature is strongly dependent on the coupling of the laser with the sample, especially at lower temperatures. We estimate an uncertainty of at least $\pm$10% of the nominal temperature in the laser-heated region depending on the focus of the laser beam, based on typical 2D temperature-gradient determination experiments performed in DACs.[9]

*d.spahr@kristall.uni-frankfurt.de

1.4. Single crystal synchrotron X-ray diffraction

Single crystal synchrotron X-ray diffraction experiments had been carried out on two different beam lines. The crystal structure of $Li_2[C_2O_5]$ was investigated at the synchrotron PETRA III (DESY) in Hamburg, Germany, at the extreme conditions beamline P02.2.[10] The beam size on the sample was $\approx 2 \times 2$ μm^2 (FWHM), focused by Kirkpatrick Baez mirrors. The diffraction data were collected using a Perkin Elmer XRD1621 detector, a wavelength of 0.2900 Å (42.7 keV) and a detector to sample distance of 402 mm. We rotated the DAC by $\pm 33°$ around the vertical axis perpendicular to the beam while collecting frames in 0.5° steps with 8 s acquisition time per frame.

Single crystal diffraction data on $Li[HC_2O_5]$ were obtained at the ESRF in Grenoble, France, at the high-pressure beam line ID27.[11] The beam size on the sample was $\approx 2 \times 2$ μm^2 (FWHM), focused by Kirkpatrick Baez mirrors. The diffraction data were collected using an Eiger2 X 9M CdTe detector, a wavelength of 0.3738 Å (33.2 keV) and a detector to sample distance of 183 mm. We rotated the DAC by $\pm 34°$ around the vertical axis perpendicular to the beam while collecting frames in 0.5° steps with 5 s acquisition time per frame.

The detector to sample distance was calibrated from the powder diffraction of a CeO_2 standard and using the software DIOPTAS.[12] The diffractometer/detector geometry for the analysis of the single crystal diffraction data was calibrated using diffraction data collected from a single crystal of enstatite ($MgSiO_3$) in a DAC at ambient pressure. After the data collection, the reflections were indexed and integrated employing CrysAlis^{PRO} (version 43.67a).[13] We used the Domain Auto Finder program (DAFi) to find possible single crystal domains for the subsequent data reduction.[14] The structure solution and refinement were performed using the software package OLEX2 employing SHELXT and SHELXS for the crystal structure determination and SHELXL for the structure refinement.[15–18]

1.5. Density functional theory-based calculations

First-principles calculations were carried out within the framework of density functional theory (DFT), employing the Perdew-Burke-Ernzerhof (PBE) exchange-correlation functional and the plane wave/pseudopotential approach implemented in the CASTEP simulation package.[19–21] "On the fly" norm-conserving or ultrasoft pseudopotentials generated using the descriptors in the CASTEP data base were employed in conjunction with plane waves up to a kinetic energy cutoff of 1020 eV or 630 eV, for norm-conserving and ultrasoft pseudopotentials, respectively. The accuracy of the pseudopotentials is well established.[22] A Monkhorst-Pack grid was used for Brillouin zone integrations.[23] We used a distance between grid points of <0.023 Å^{-1}. Convergence criteria for geometry optimization included an energy change of $<5 \times 10^{-6}$ eV atom^{-1} between steps, a maximal force of <0.008 eV Å^{-1} and a maximal component of the stress tensor <0.02 GPa. Phonon frequencies were obtained from density functional perturbation theory (DFPT) calculations.[24,25] Raman intensities were computed using DFPT with the "$2n + 1$" theorem approach.[26] A correction scheme for van der Waals (v.d.W.) interactions was applied for the DFT-calculations. We employed the correction scheme developed by Tkatchenko and Scheffler.[27]

2. Results

2.1. *Single crystal synchrotron X-ray diffraction on $Li_2[C_2O_5]$*

Due to the identification of both unknown phases by Raman spectroscopy, we were able to perform synchrotron X-ray diffraction experiments in the regions where mainly Raman modes of one of the unknown phases have been observed in the DAC. The experiments had been carried out at the synchrotron PETRA III. We found that the first unknown phase is a lithium pyrocarbonate with $Li_2[C_2O_5]$ composition. After the data reduction the crystal structure was solved in the monoclinic space group $P2_1/c$ (No. 14) with $Z = 4$. Due to the metallic body of the DAC the access to the reciprocal space is very limited. Nevertheless, the displacement parameters of all atoms could be refined anisotropically and no constraints or restraints had to been introduced, even if only light atoms are present. We reached a stable crystal structure refinement with a reasonable R-value (5.8%), but the reflection to parameter ratio is relatively low (6.6:1). Table S 1 lists the crystallographic parameters of $Li_2[C_2O_5]$ valid for 25(2) GPa in comparison to DFT calculations.

Table S 1: Structural parameters of $Li_2[C_2O_5]$ at 25(2) GPa from single crystal structure solution (ambient temperature) in comparison to theoretical data derived from DFT calculations (athermal limit).

	Single Crystal	DFT
**Crystal data**		
Crystal system	Monoclinic	Monoclinic
Space group	$P2_1/c$	$P2_1/c$
Chemical formula	$Li_2[C_2O_5]$	$Li_2[C_2O_5]$
M_r	117.9	117.9
a (Å)	6.085(1)	6.0611
b (Å)	5.313(3)	5.2206
c (Å)	7.996(3)	7.8542
α (°)	90.0	90.0
β (°)	100.85(3)	101.02
γ (°)	90.0	90.0
V (Å^3)	253.9(2)	243.95
Z	4	4
**Data collection**		-
F_{000}	232	-
θ range (°)	1.89–12.88	-
measured reflections	887	-
independent reflections	537	-
reflections $I > 2\sigma(I)$	406	-
R_{int}	0.033	-
		-
**Refinement**		-
$R[F^2 > 2\sigma(F^2)]$, $wR(F^2)$	0.058, 0.153	-
No. of reflections	537	-
No. of parameters	82	-
No. of restraints	0	-
No. of constraints	0	-
$\Delta\rho_{max}$, $\Delta\rho_{min}$ (e Å^{-3})	0.60, −0.56	-

The experimental and the theoretical structural model of $Li_2[C_2O_5]$ match within the expected errors. Our DFT calculations confirmed the centrosymmetric space group symmetry. Due to the presence of only light elements (lithium, oxygen and carbon) the intensity of the diffraction data is relatively low. In $Li_2[C_2O_5]$ both of the lithium atoms are octahedrally coordinated by six oxygen atoms (Fig. S 1). Both octahedra are distorted with Li–O bond distances between 1.8 Å and 2.2 Å.

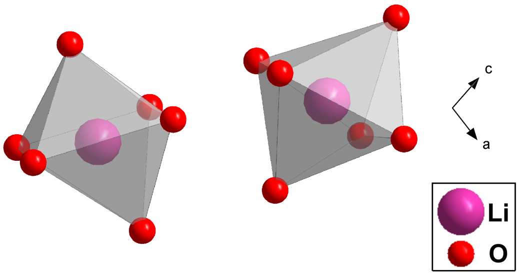

Figure S 1: Octahedral coordination of both lithium atoms in $Li_2[C_2O_5]$ by six oygen atoms. The coordination polyhedra are shown in grey.

2.2. *Single crystal synchrotron X-ray diffraction on $Li[HC_2O_5]$*

After the identification and structure solution of the first unknown phase $Li_2[C_2O_5]$, we performed synchrotron X-ray diffraction experiments in the region where the second unknown phase was observed by Raman spectroscopy. These diffraction experiments had been performed at the synchrotron ESRF. The results from the single crystal structure solution show that this phase is a lithium hydrogen pyrocarbonate with $Li[HC_2O_5]$ composition. The crystal structure was solved in the monoclinic space group $C2/c$ (No. 15) with $Z = 4$. The hydrogen atom was directly recognized as a Fourier difference peak. We assume a reasonable structure refinement due to the relatively low R-value (4.9%) and a sufficient reflection to parameter ratio (7.5:1). The displacement parameters of the lithium, carbon and oxygen atoms were refined anisotropically, while the one from hydrogen atom was refined isotropically. In order to reduce the amount of parameters the anisotropic displacement of the oxygen and carbon atoms were constraint to be identical, respectively. No constraints or restraints for the atomic positions had been introduced.

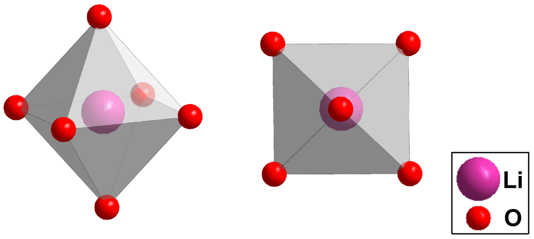

Figure S 2: Octahedral coordination of the lithium atom in $Li[HC_2O_5]$ by six oxygen atoms viewed in two different directions. The coordination polyhedra are shown in grey.

The experimental crystallographic parameters of $Li[HC_2O_5]$ are listed in Table S 2 in comparison to DFT calculations. The DFT calculations had been carried out

in the non-centrosymmetric monoclinic space group *Cc* (No. 9) to ensure that no symmetry restrictions were applied to the position of the hydrogen atom. Nevertheless, the theoretical structural model of Li[HC_2O_5] reproduces the experimental one within the expected errors. A symmetry analysis shows, that after geometry optimization, the structural model from the DFT calculations has space group symmetry $C2/c$. The space group symmetry of geometry-optimized structures were analyzed using Materials Studio.[28] The results obtained by a refinement of the experimental diffraction data in space group *Cc* cannot be distinguished from the structural model having $C2/c$ space group symmetry. Hence, we decided to use the centrosymmetric structural model. In Li[HC_2O_5] the lithium atom is coordinated by six oxygen atoms, similar to Li_2[C_2O_5] (Fig. S 2). In contrast to Li_2[C_2O_5] the octahedra in Li[HC_2O_5] is nearly undistorted with Li–O bond distances between 1.9 Å and 2.0 Å.

Table S 2: Structural parameters of Li[HC_2O_5] at 25(2) GPa from single crystal structure solution (ambient temperature) in comparison to theoretical data derived from DFT calculations (athermal limit).

	**Single Crystal**	**DFT**
**Crystal data**		
Crystal system	Monoclinic	Monoclinic
Space group	$C2/c$	Cc*
Chemical formula	Li[HC_2O_5]	Li[HC_2O_5]
M_r	111.97	111.97
a (Å)	12.085(9)	11.6312
b (Å)	4.373(1)	4.3655
c (Å)	5.231(7)	5.2071
α (°)	90.0	90.0
β (°)	117.5(1)	114.15
γ (°)	90.0	90.0
V (Å^3)	245.3(4)	241.27
Z	4	4
**Data collection**		-
F_{000}	224	-
θ range (°)	2.65–21.48	-
measured reflections	349	-
independent reflections	231	-
reflections $I > 2\sigma(I)$	178	-
R_{int}	0.005	-
		-
**Refinement**		-
$R[F^2 > 2\sigma(F^2)]$, $wR(F^2)$	0.049, 0.149	-
No. of reflections	231	-
No. of parameters	31	-
No. of restraints	0	-
No. of constraints	2	-
$\Delta\rho_{max}$, $\Delta\rho_{min}$ (e Å^{-3})	0.34, −0.23	-

*symmetry analysis after geometry optimization suggests space group $C2/c$

It is worthwhile to note here, that the cryogenic loading was performed with Li_2[CO_3] and CO_2, but nevertheless hydrogen was found in the structure solution. As mentioned above we think that small amounts of H_2O were inadvertently co-condensed with the CO_2 gas jet during the cryogenic loading, as Li_2[CO_3] is not particularly hygroscopic and no hydrous Li-carbonate is known.

2.3. *Bulk modulus of Li[HC_2O_5] and Li_2[C_2O_5]*

We used DFT-based calculations to calculate the p, V relation for Li[HC_2O_5]. The calculations were carried out between 0 GPa and 50 GPa. One data-set was calculated without a v.d.W. correction and the second data-set was obtained using the TS-v.d.W. correction scheme.[27] We found that down to ≈ 4 GPa no significant difference between both theoretical data-sets can be observed (Fig. S 3). Below this pressure, the unit cell volume obtained without a v.d.W. correction is significantly too large.

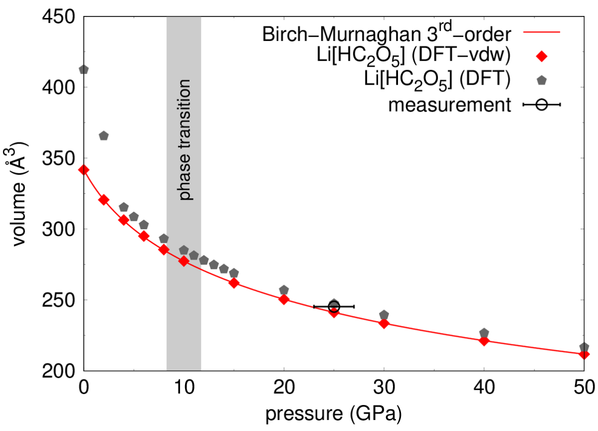

Figure S 3: A Birch-Murnaghan EoS was fitted to the unit cell volume obtained from the v.d.W.-corrected DFT calculations of Li[HC_2O_5] between 0–50 GPa. In addition DFT data without a v.d.W. correction are shown. The result from the single crystal structure solution is shown for comparison.

We fitted a 3rd-order Birch-Murnaghan equation of states (EoS) to p, V-data derived from the calculations to obtain the theoretical bulk modulus (K_0).[29,30] We used the software EOSFit7-GUI.[31] First we fitted the p, V date in the pressure range above the phase transition (10–50 GPa), present due to the change of the hydrogen position and the O–H··O geometry. We found that no significant difference occurs if the EoS is fitted over the whole pressure range (0–50 GPa), see Table S 3. We obtained a bulk modulus of $K_0 = 25.7(4)$ GPa with $K_p = 5.9(1)$.

Table S 3: Theoretical bulk modulus of Li[HC_2O_5] and Li_2[C_2O_5] obtained by fitting a Birch-Murnaghan EoS to the p, V data from the DFT-based calculations.

	V_0 (Å^3)	K_0 (GPa)	K_p
Li[HC_2O_5] (0–50 GPa)	342.4(4)	25.6(4)	5.93(5)
Li[HC_2O_5] (10–50 GPa)	342.1(6)	25.7(4)	5.94(4)
Li_2[C_2O_5] (10–50 GPa)	318.3(8)	43(1)	5.9(1)

In addition, we used DFT-based calculations to obtain the p, V relation for $Li_2[C_2O_5]$ employing a v.d.W. correction. Again, the calculations were carried out between 0 GPa and 50 GPa, but the results show two discontinuities in the behavior of the unit cell volume. We assume that at low pressures two phase transitions occur in $Li_2[C_2O_5]$. Hence we fitted the 3rd-order Birch-Murnaghan EoS in the pressure range above these phase transition (10–50 GPa) to the theoretical data to obtain the bulk modulus (Fig. S 4). We obtained a bulk modulus of $K_0 = 43(1)$ GPa with $K_p = 5.9(1)$ for $Li_2[C_2O_5]$, which is significantly larger than for hydrogen pyrocarbonate in the same pressure range (Table S 3).

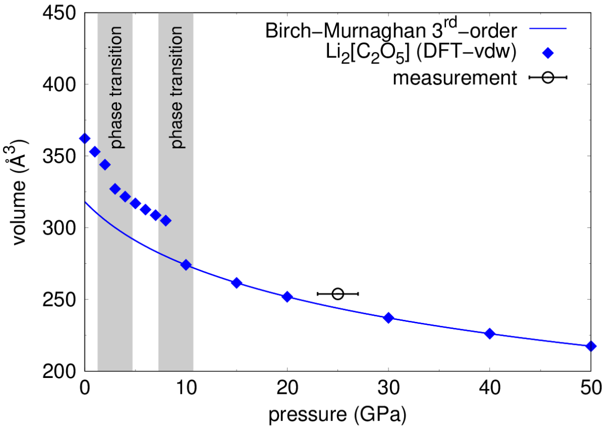

Figure S 4: A Birch-Murnaghan EoS was fitted to the unit cell volume obtained from the v.d.W.-corrected DFT calculations of $Li_2[C_2O_5]$ between 10–50 GPa. Low pressure p, V data were not considered for the fit. The result from the single crystal structure solution is shown for comparison.

2.4. *Phase transition in* $Li_2[C_2O_5]$

We used the DFT calculations to investigate the phase transitions of $Li_2[C_2O_5]$ at lower pressures. We found that both low-pressure phases have monoclinic $P2_1/c$ space group symmetry and that the transitions are accompanied by a change of the monoclinic angle β (Fig. S 5).

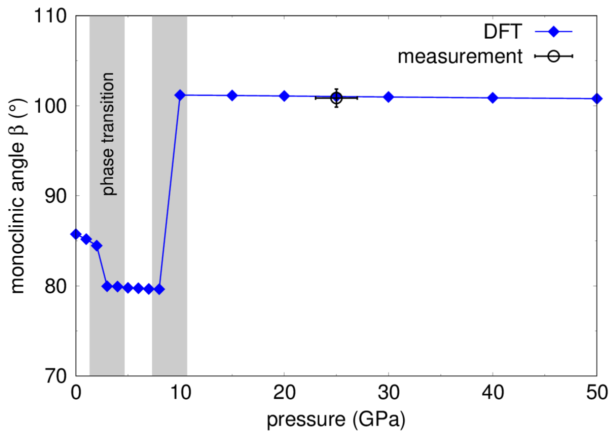

Figure S 5: Monoclinic angle β between 0 GPa and 50 GPa from DFT calculations. Two phase transitions at pressures < 10 GPa are indicated by grey bars. The result from the single crystal structure solution is shown for comparison.

At pressures $\geq$ 10 GPa the monoclinic angle β is in very good agreement with the results from the single crystal structure solution of the high-pressure phase at 25(2) GPa, while at lower pressures β decreases strongly. In addition, the phase transitions cause a visible change in the geometry of the $[C_2O_5]^{2-}$-groups (Fig. S 6).

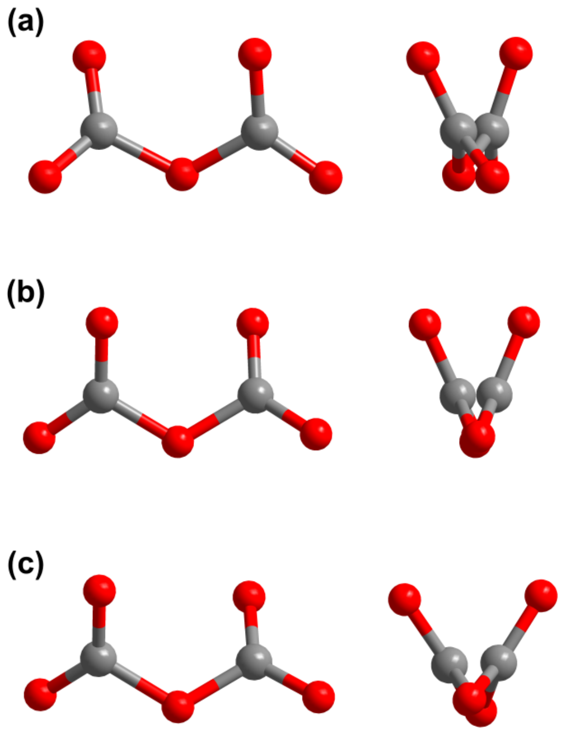

Figure S 6: Geometry of the $[C_2O_5]^{2-}$-groups in $Li_2[C_2O_5]$ at (a) 10 GPa, (b) 5 GPa and (c) 0 GPa.

References

(1) Boehler, R. New diamond cell for single–crystal X-ray diffraction. *Rev. Sci. Instrum.* **2006**, *77*, 115103–1–115103–3, DOI: 10.1029/JB091iB05p04673

(2) Mao, H. K.; Xu, J.; Bell, P. M. Calibration of the ruby pressure gauge to 800 kbar under quasi-hydrostatic conditions. *J. Geophys. Res.* **1986**, *91*, 4673–4676, DOI: 10.1029/JB091iB05p04673

(3) Yoo, C. S.; Cynn, H.; Gygi, F.; Galli, G.; Iota, V.; Nicol, M.; Carlson, S.; Häusermann, D.; Mailhiot, C. Crystal Structure of Carbon Dioxide at High Pressure: "Superhard" Polymeric Carbon Dioxide. *Phys. Rev. Lett.* **1999**, *83*, 5527–5530, DOI: 10.1103/PhysRevLett.83.5527

(4) Spahr, D.; König, J.; Bayarjargal, L.; Luchitskaia, R.; Milman, V.; Perlov, A.; Liermann, H.-P.; Winkler, B.; Synthesis and Structure of Pb[C_2O_5]: An Inorganic Pyrocarbonate Salt. *Inorg. Chem.* **2022**, *61*, 9855–9859, DOI: 10.1021/acs.inorgchem.2c01507

(5) Scelta, D.; Ceppatelli, M.; Ballerini, R.; Hajeb, A.; Peruzzini, M.; Bini, R. Sprayloading: A cryogenic deposition method for diamond anvil cell. *Rev. Sci. Instrum.* **2018**, *89*, 053903, DOI: 10.1063/1.5011286

(6) Bayarjargal, L.; Fruhner, C.-J.; Schrodt, N.; Winkler, B. $CaCO_3$ phase diagram studied with Raman spectroscopy at pressures up to 50 GPa and high temperatures and DFT modeling. *Phys. Earth Planet. Inter.* **2018**, *281*, 31–45, DOI: 10.1016/j.pepi.2018.05.002

(7) Wojdyr, M. *Fityk*: a general-purpose peak fitting program. *J. Appl. Cryst.* **2010**, *43*, 1126–1128, DOI: 10.1107/S0021889810030499

(8) Benedetti, L. R.; Loubeyre, P. Temperature gradients, wavelength-dependent emissivity, and accuracy of high and very-high temperatures measured in the laser-heated diamond cell. *High Press. Res.* **2004**, *24*, 423–455, DOI: 10.1080/08957950412331331718

(9) Du, Z.; Amulele, G.; Benedetti, L. R.; Lee, K. K. M. Mapping temperatures and temperature gradients during flash heating in a diamond-anvil cell. *Rev. Sci. Instrum.* **2013**, *84*, 075111, DOI: 10.1063/1.4813704

(10) Liermann, H.-P.; Konôpková, Z.; Morgenroth, W.; Glazyrin, K.; Bednarčik, J.; McBride, E. E.; Petitgirard, S.; Delitz, J. T.; Wendt, M.; Bican, Y.; Ehnes, A.; Schwark, I.; Rothkirch, A.; Tischer, M.; Heuer, J.; Schulte-Schrepping, H.; Kracht, T.; Franz, H. The Extreme Conditions Beamline P02.2 and the Extreme Conditions Science Infrastructure at PETRAIII. *J. Synchrotron Radiat.* **2014**, *22*, 908–924, DOI: 10.1107/S1600577515005937

(11) Mezouar, M.; Garbarino, G.; Bauchau, S.; Morgenroth, W.; Martel, K.; Petitdemange, S.; Got, P.; Clavel, C.; Moyne, A.; Van Der Kleij, H.-P.; Pakhomova, A.; Wehinger, B.; Gerin, M.; Poreba, T.; Rosa, A.; Forestier, A.; Weck, G.; Datchi, F.; Wilke, M.; Jahn, S.; Andrault, D.; Libon, L.; Pennacchioni, L.; Laniel, D.; Bureau H. The high flux nano-X-ray diffraction, fluorescence and imaging beamline ID27 for science under extreme conditions on the ESRF Extremely Brilliant Source. *High Press. Res.* **2024**, *44*, 171–198, DOI: 10.1080/08957959.2024.2363932

(12) Prescher, C.; Prakapenka, V. B. *DIOPTAS*: a program for reduction of two-dimensional X-ray diffraction data and data exploration. *High. Press. Res.* **2015**, *35*, 223–230, DOI: 10.1080/08957959.2015.1059835

(13) Agilent, CrysAlis PRO, Yarnton, England, **2014**

(14) Aslandukov, A.; Aslandukov, M.; Dubrovinskaia, N.; Dubrovinsky, L. *Domain Auto Finder* (*DAFi*) program: the analysis of single-crystal X-ray diffraction data from polycrystalline sample. *J. Appl. Cryst.* **2022**, *55*, 1383–1391, DOI: 10.1107/S1600576722008081

(15) Dolomanov, O. V.; Bourhis, L. J.; Gildea, R. J.; Howard, J. A. K.; Puschmann, H. *OLEX2*: a complete structure solution, refinement and analysis program. *J. Appl. Cryst.* **2009**, *42*, 339–341, DOI: 10.1107/S0021889808042726

(16) Sheldrick, G. M. *SHELXT* — Integrated space-group and crystal-structure determination. *Acta. Cryst.* **2015**, *A71*, 3–8, DOI: 10.1107/S2053273314026370

(17) Sheldrick, G. M. A short history of *SHELX*. *Acta. Cryst.* **2008**, *A64*, 112–122, DOI: 10.1107/S0108767307043930

(18) Sheldrick, G. M. Crystal structure refinement with *SHELXL*. *Acta. Cryst.* **2015**, *C71*, 3–8, DOI: 10.1107/S2053229614024218

(19) Hohenberg, P.; Kohn, W. Inhomogeneous Electron Gas. *Phys. Rev.* **1967**, *136*, B864–B871, DOI: 10.1103/PhysRev.136.B864

(20) Perdew, J. P.; Burke, K.; Ernzerhof, M. Generalized Gradient Approximation Made Simple. *Phys. Rev. Lett.* **1996**, *77*, 3865–3868, DOI: 10.1103/PhysRevLett.77.3865

(21) Clark, S. J.; Segall, M. D.; Pickard, C. J.; Hasnip, P. J.; Probert, M. I. J.; Refson, K.; Payne, M. C. First principles methods using CASTEP. *Z. Kristallogr.* **2005**, *220*, 567–570, DOI: 10.1524/zkri.220.5.567.65075

(22) Lejaeghere, K.; Bihlmayer, G.; Björkman, T.; Blaha, P.; Blügel, S.; Blum, V.; Caliste, D.; Castelli, I. E.; Clark, S. J.; Dal Corso, A. et al. Reproducibility in density functional theory calculations of solids. *Science* **2016**, *351*, aad3000, DOI: 10.1126/science.aad3000

(23) Monkhorst, H. J.; Pack, J. D. Special points for Brillouin-zone integrations. *Phys. Rev. B* **1976**, *13*, 5188–5192, DOI: 10.1103/PhysRevB.13.5188

(24) Baroni, S.; de Gironcoli, S.; Dal Corso, A.; Giannozzi, P. Phonons and related crystal properties from density-functional perturbation theory. *Rev. Mod. Phys.* **2001**, *73*, 515–562, DOI: 10.1103/RevModPhys.73.515

(25) Refson, K.; Tulip, P. R.; Clark, S. J. Variational density-functional perturbation theory for dielectrics and lattice dynamics. *Phys. Rev. B* **2006**, *73*, 155114, DOI: 10.1103/PhysRevB.73.155114

(26) Miwa, K. Prediction of Raman spectra with ultrasoft pseudopotentials. *Phys. Rev. B* **2011**, *84*, 094304, DOI: 10.1103/PhysRevB.84.094304

(27) Tkatchenko, A.; Scheffler, M. Accurate Molecular Van Der Waals Interactions from Ground-State Electron Density and Free-Atom Reference Data. *Phys. Rev. Lett.* **2009**, *102*, 073005, DOI: 10.1103/PhysRevLett.102.073005

(28) Meunier, M.; Robertson, S. *Materials Studio* 20th anniversary. *Mol. Simul.* **2021**, *47*, 537–539, DOI: 10.1080/08927022.2021.1892093

(29) Murnaghan, F. The Compressibility of Media under Extreme Pressures. *Proc. Natl. Acad. Sci.* **1944**, *30*, 244–247, DOI: 10.1073/pnas.30.9.244

(30) Birch, F. Finite Elastic Strain of Cubic Crystals. *Phys. Rev.* **1947**, *71*, 809–824, DOI: 10.1103/PhysRev.71.809

(31) Gonzalez-Platas, J.; Alvaro, M.; Nestola, F.; Angel, R. *EosFit7-GUI*: a new graphical user interface for equation of state calculations, analyses and teaching. *J. Appl. Cryst.* **2016**, *49*, 1377–1382, DOI: 10.1107/S1600576716008050
